# Le don de gamètes dans la prise en charge de l’infertilité du couple: connaissances, attitudes et perception des étudiants en médecine de la Faculté des Sciences de la Santé de Cotonou-Bénin

**DOI:** 10.11604/pamj.2022.41.147.26430

**Published:** 2022-02-18

**Authors:** Simon Azonbakin, Ulrich Gahou, Flore Gangbo, Patrice Dangbemey, Diane Adovoekpe

**Affiliations:** 1 Unité de Biologie Humaine, Faculté des Sciences de la Santé, Cotonou, Benin,; 2 Centre Mère-Enfant, Faculté des Sciences de la Santé, Université d´Abomey-Calavi, Cotonou, Benin

**Keywords:** Couple infertile, assistance médicale à la procréation, don de gamètes, Infertile couple, medically assisted procreation, gamete donation

## Abstract

**Introduction:**

de nos jours l´infertilité est un réel problème de santé publique. Parmi les solutions proposées il y a l´assistance médicale à la procréation (AMP) avec le don de gamètes. Au Benin, malgré un cadre législatif bien défini (code des enfants), le don de gamètes en vue d´une AMP, n´a pas fait l´objet d´une étude. Nous nous proposons d´étudier des connaissances, attitudes et perceptions des étudiants en médecine de la FSS de Cotonou par rapport au don de gamètes.

**Méthodes:**

une étude transversale et descriptive a été réalisée auprès des étudiants du 2^e^ et 3^e^ cycle en médecine à la Faculté des Sciences de la Santé (FSS) de Cotonou au Bénin.

**Résultats:**

au total 236 étudiants dont 54% (n=127) d´homme et 46% (n= 109) de femme. L´âge moyen était de 23 ans. La connaissance du traitement par l´AMP avec recours au don de gamètes est de 90,6%, (n=214). La possibilité de ce traitement au Bénin est connue de 55,6% (n=131). Un peu plus de quatre vingt-huit pour cent (n=209) des enquêtés ignorent l´existence d´une législation en vigueur depuis 2015 sur la question. Plus, de soixante-neuf pour cent (n=69%) (n=164) refusent de faire don des gamètes. La raison éthique était principalement évoquée.

**Conclusion:**

la connaissance des étudiants sur l´AMP par le don de gamètes est correcte et leur refus de faire don de leurs gamètes est estimée importante avec comme raison dominante de refus l´éthique.

## Abstract (en)

**Introduction:**

infertility is a real public health problem. Medically Assisted Procreation (MAP) with donor gametes is one of the possible solutions. In Benin, despite a well-defined legislative framework (children´s code), MAP using donated gametes has not been studied. The purpose of this study was to assess knowledge, attitudes and perceptions of students at the Faculty of Health and Medical Sciences in Cotonou about the donation of gametes.

**Methods:**

we conducted a cross-sectional and descriptive study among second or third-year medical students at the Faculty of Health and Medical Sciences (FHMS) in Cotonou.

**Results:**

the surveyed sample consisted of 236 students, of whom 54% (n=127) were male and 46% (n=109) were female; the representative age range was between 18 and 24 years (87%). The knowledge of MAP using donated gametes was 90.6% (n=214). The possibility that this treatment might be performed in Benin was known to be 55.6% (n=131). More that eighty-eight percent (n=209) of respondents were unaware of the existence of legislation in force since 2015 on this matter and 69.5% (n=164) refused to donate gametes. Among the reasons mentioned, the ethical reason dominated.

**Conclusion:**

in Benin, students´ knowledge about MAP using donated gametes is adequate but there is a widespread refusal to donate their gametes among them, mainly because of ethical issues.

## Introduction

L´infertilité est définie comme l´incapacité pour une personne d´être l´auteur d´une grossesse en considérant que son partenaire est normal sur le plan de la fertilité [1]. Ainsi l´infertilité du couple est définie par l´Organisation Mondiale de la Santé (OMS) comme étant l´absence de grossesse chez un couple en âge de procréer (femme âgée de 18 à 45 ans) au bout de 12 mois de rapports sexuels réguliers, fréquents et sans contraception [2]. Il est actuellement admis que bon nombre de centres pratiquant l´AMP font recours au don de gamètes compte tenu des législations en vigueur dans chaque pays. Car les patients qui y recourent ont souvent des altérations sévères de la production et de survie de gamète [3]. C´est le cas des asthénozoospermies sévères, ou bien des cas d´azoospermie chez l´homme [4]. Chez la femme, depuis quelques années, les indications du don d´ovocytes se sont élargies aux insuffisances ovariennes débutantes ou aux échecs de fécondation, qui concernent majoritairement des femmes de plus de 38 ans. Aussi, le don de gamètes permet de prévenir la transmission d´une anomalie génétique particulièrement létale chez l´enfant. Les enfants issus de ce don, sont des enfants du couple et l´anonymat est maintenu pour le don [5]. Dans les pays développés ceci est bien encadré par la loi. Les couples qui y font recours l´assurent pleinement [6]. En Afrique subsaharienne, le Bénin est l´un des rares pays à disposer d´une législation qui autorise et encadre l´AMP avec le don des gamètes. Cet arsenal juridique peu connu et les cultures traditionnelles, très natalistes ne permettent pas de bien intégrer la nécessité du don de gamètes afin d´avoir un enfant. Le mariage étant vu comme un vecteur de continuation de la vie clanique. Plusieurs patients auraient des notions sur le don de rein, de foie et d´autres organes surtout le don de sang [7-9]. Mais en ce qui concerne le don de gamètes, cette problématique n´a jamais été abordée en population béninoise. Notre étude aborde pour la première fois la problématique de don de gamètes au sein de la population béninoise et probablement au sein de la population ouest africaine. Nous nous proposons alors dans cette étude, d´explorer les connaissances, attitudes et perceptions des étudiants en médecine de la Faculté des Sciences de la Santé (FSS) par rapport à cette problématique de don de gamètes.

## Méthodes

**Conception et cadre de l´étude:** il s´agit d´une étude transversale et descriptive réalisée de novembre 2017 à juin 2018, à travers un sondage d´opinion effectué à l’unité de formation et de recherche en médecine à la Faculté des Sciences de la Santé de l´Université d´Abomey-Calavi, Bénin. L´étude a été menée dans le but d´évaluer les connaissances attitudes et pratiques des étudiants en médecine par rapport à l´AMP avec don de gamètes.

**Population de l´étude:** tous les étudiants du 2^e^ ou 3^e^ cycle des études médicales de la Faculté des Sciences de la Santé qui ont déjà acquis des notions générales en anatomie, physiologie et physiopathologie des appareils génitaux ont été inclus. Les étudiants ayant refusé de participer à l´étude, ainsi que ceux qui n´ont pas validé toutes les unités de formation permettant d´être au second cycle n´ont pas été inclus.

**Recueils de données:** la collecte des données a été effectuée au moyen d´une fiche initialement conçue et testée à l´avance sur la base d´une grille conçue à cet effet.

**Analyse des données:** les données ont été saisies, codifiées et analysées grâce à une maquette confectionnée sur le logiciel IBM SPSS Statistics version 2.1. Le calcul des fréquences simple a été utilisé pour la description de l´échantillon. L´association entre les variables a été déterminée par leur croisement à l´aide du test de Khi carré de Karl Pearson au seuil de signification de 5%. Le rapport de prévalence (p-value) a permis de déterminer le degré d´association. La rédaction du rapport et l´organisation des données sous forme de tableaux ont été réalisées avec les logiciels Word et Excel 2010.

**Considérations éthiques:** la présente étude, réalisée dans le cadre des travaux académiques, a été conduite dans le strict respect des règles de bonnes pratiques cliniques (BPC). Le consentement libre et éclairé des patients a été obtenu. La confidentialité a été rigoureusement respectée au cours de la collecte des données. Les informations obtenues dans le cadre de cette étude ont été traitées dans l'anonymat. les participants, ont été informés que leur participation à l´étude est totalement volontaire, qu´ils peuvent se retirer de l´étude à tout moment.

## Résultats

### Aspects socio-épidémiologiques

Deux-cent trente-six étudiants ont participé à l´enquête dont 54% (n=127) de sexe masculin et 46% (n=109) de sexe féminin soit un sexe ratio de 1,2. La couche d´âge la plus représentée est celle des étudiants ayant entre 18 et 24 ans avec un âge moyen de 23 ans. Les étudiants de la 6^e^ année étaient les plus nombreux. En ce qui concerne la religion, les chrétiens étaient les plus importants (87%, n=205).

### Connaissances des répondants

**Le niveau de connaissances des répondants se présente comme il suit:** la definition de l´infertilité. Quatre-vingt huit pourcent (n=207) des participants adhèrent à la nouvelle définition de l´infertilité de l´Organisation Mondiale de la Santé (OMS) selon laquelle «l´infertilité du couple est définie comme étant l´absence de grossesse chez un couple en âge de procréer (femme âgée de 18 à 45 ans) au bout de 12 mois de rapports sexuels fréquents, réguliers sans contraception».

**La source d´information:** dans notre étude, 55,9% des participants affirment avoir reçu l´information relative à l´AMP pendant les cours théoriques et/ou pratiques, 61% dans les mass médias et 15,7% d´autres sources comme les causeries amicales, l´internet etc.

**L´aspect législatif sur le don de gamètes:** pour 88,41% (n=206) des participants, il n´existe pas au Bénin une législation sur l´AMP et le don de gamètes (Tableau 1). En croisant les données relatives à l´année d´étude et l´existence au Bénin d´une législation, les participants de la sixième année pensent en grande partie que cette législation existe. Ainsi, il existerait une dépendance entre ces deux variables (p = 0,026).

**Tableau 1:** existence au Bénin d’une législation sur l’AMP et le don de gamètes

	Effectifs	Pourcentage
Oui	27	11,40
Non	206	87,41
Ne sait pas	3	0,19
**Total**	**236**	**100,0**

**Différentes causes d´infertilité nécessitant le recours au don de gamètes:** pour ce qui est des pathologies nécessitant le recours au don de gamètes, la majorité des patients n´avaient pas pu citer les causes de l´infertilité nécessitant le recours au don de gamètes (82,6%, n=195).

### Attitudes et perception des répondants

**Figure 1:**
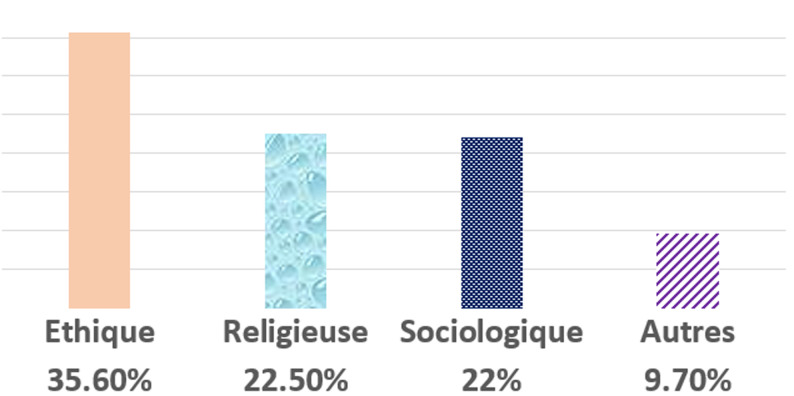
répartition des raisons du refus de don de gamètes

**Figure 2:**
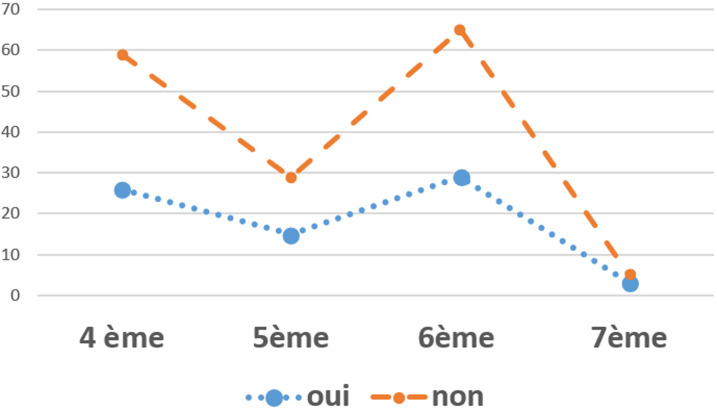
relation entre l´année d'étude en médecine et l´acceptation du don de gamètes

Dans notre étude, 31,5% (n=74) des participants acceptent de faire le don de gamètes. Les raisons de refus sont: éthique, religieux, sociologique et autres) (Figure 1). Pour 49,8% (n=118) des participants les enfants issus du don de spermatozoïdes et d´ovocytes sont des enfants légitimes. En croisant les données, on a note qu´il existerait un lien statistiquement significatif entre l´âge et l´acceptation du don des gamètes (p = 0,045) (Tableau 2). Aussi, a t-on remarqué qu´il existe un lien entre le sexe et l´acception de don de gamètes; les hommes ayant plus tendance à donner leur gamète (Tableau 3). La proportion des participants qui acceptent de faire le don de gamètes varie d´une année d´étude à une autre: on observe une diminution de refus au don de gamètes d´environ 4% entre la quatrième année et la septième année d´étude (Figure 2). En ce qui concerne la religion des repondants, il n´avait pas de lien statistiquement significatif entre la religion et le don de gamètes. Afin d´assurer la promotion et la diffusion du don de gamètes dans le cadre de l´AMP, 70,8% (n=167) des étudiants estiment qu´il faudrait mettre en place des associations de couples infertiles afin d´ouvrir le débat au grand public via les medias.

**Tableau 2:** relation entre l’âge et l’acceptation de faire le don des gamètes

		Accepter faire don des gamètes pour aider		Total
		Oui	Non	
**Classe d´âge**	18-24	29,6%	70,4%	100,0%
	25 et plus	48,3%	51,7%	100,0%
**Total**		**32,1%**	**6,9%**	**100,0%**

**Tableau 3:** relation entre le sexe et l´acceptation du don de gamètes

		Accepter faire don des gamètes		Total
		Oui	Non	
Sexe	**Masculin**	37,8%	62,2%	100,0%
	**Féminin**	23,8%	76,2%	100,0%
**Total**		**31,5%**	**68,5%**	**100,0%**

## Discussion

La prise en charge de l´infertilité grace à l´AMP avec le don de gamètes n´ayant pas encore fait l´objet d´études au Bénin, nous nous sommes proposés d´étudier les connaissances, attidtudes et perception des étudiants en Médécine de Cotonou par rapport à cette problématique. La definition harmonisée de l´OMS, notamment pour ce qui concerne la durée de l´infertilité a été discutée par bon nombre d´étudiants. Aussi, selon certains auteurs, la définition actualisée de l´infertilité de l´OMS pose un problème, dans la mesure où le délai de 12 mois s´est avéré très court. Jusqu´en 2008, l´infertilité se mesurait sur une période de vingt-quatre mois, et non pas de douze mois, comme le suggère la nouvelle définition de l´OMS [1,2]. Un rapport de l´ESHRE (European Society of Human Reproduction and Embryology) montre que seulement 9% des couples demeurent infertiles après 12 mois ou plus. Or, à ce jour, aucun moyen n´aurait été mis en place pour vérifier que le délai d´un an d´essais infructueux a bien été écoulé avant d´entreprendre le premier cycle de l´AMP [10]. Aussi, la définition de l´infertilité s´envisage désormais pour une personne et non un couple quel que soit le sexe [11].

Au Bénin, le code de l´enfant prévoit les conditions pour le don de gamètes, les critères médicaux du donneur ainsi que les clauses de l´AMP. En Afrique noire francophone, le Bénin serait probablement le seul pays a disposé d´une telle législation. Cette loi n´est pas connue de la majorié des enquetés. Cette méconnaissance de la législation tout comme beaucoup d´autres textes de loi se pose dans le pays. Dans d´autres pays, la loi n´est pas explicite en ce qui concerne l´AMP avec don de gamètes. Selon Pierre Léon André DIENG de l´Université Cheikh Anta DIOP de Dakar, « dans les pays en développement, notamment africains, asiatiques, sud-américains et arabes, la pratique de don de gamètes est vécue avec plus de pudeur, de réserve et de circonspection [13,14]. Dans la majorité des pays développés, l´AMP et le don de gamètes sont hautement encadrées par des textes de lois [15-17]. En ce qui concerne les conditions médicales nécessitant le don de gamètes, les causes génétiques rares pouvant justifié un don de gamètes en vue d´une AMP n´ont pratiquement pas été évoquées. La méconnaissance des étudiants de ces étiologies génétiques pouvant nécessiter le recours au don de gamètes pourrait s´expliquer par la tendance globale des praticiens à négliger les maladies génétiques en général devant le poids important des pathologies tropicales [18].

La question de l´anonymat du don de gamètes s´est posée: la majorité des enquete avait opté pour l´anonymat du don. Ce résultat est conforme à celui de L. Karpel *et al*. en France [19]. En effet selon cet auteur, les couples demandeurs et les patients donneurs étaient favorables à plus de 85% au don de gamètes anonyme. Quinze pourcent des couples ayant eu recours au don anonyme auraient choisi le don direct et non anonyme si cela avait été possible. Enfin, en faveur de l´anonymat, il y a la donneuse réelle: deux tiers des parents préfèrent n´en avoir aucune connaissance. Ce sont seulement les informations médicales du donneur qui les interessent [20,21].

Près de la moitié de la population estime que les enfants issus de don de spermatozoïdes et d´ovocytes sont légitimes. Cette reconnaissance de statut d´enfant traduirait l´acceptation par les populations de l´enfant issu du don. Mais cela pourrait cacher des interrogations comme les notions de parenté et de filiation [22]. Ceci est une prescription légale dans la plupart des pays autorisant le don de gamètes [20].

Seulement un tiers des répondants avait accepté de faire le don de gamètes. Ceci est faible quand on compare au don d´organe au Pakistan par exemple à 45% [8]. Il en est de même lorsqu´on considère l´acceptation du don de rein dans la population sénégalaise qui est de 71,5% en 2012 [8]. Comme l´expriment d´ailleurs les résultats sur la difficulté du don de gamètes par rapport aux autres organes, il paraît plus facile pour les étudiants de donner un organe comme le rein par exemple plutôt que de donner leurs gamètes. La majorité des répondants s´opposent au don de gamètes pour des raisons éthiques, religieux, sociologiques et personnels.

Parmi les étudiants acceptant de donner les gamètes, il y a une partie importante qui a connaissance d´un patient infertile. Ceci concorde avec les résultats d´une étude de Karpel L., réalisée en 2001, à l´Unité d´Aide Médicale à la Procréation de l´Hôpital Antoine-Béclère en France. Il a prouvé que les couples receveurs ont beaucoup motivé les proches pour le don de gamètes [19]. Pour encourager le don de gamètes au Bénin, 70,8% des répondants pensent qu´il faut informer d´avantage le public sur le nombre de patients en attente d´AMP, et apporter des témoignages. Un couple motivé pour donner ses gamètes est un couple qui a été sensibilisé, de près ou de loin à la détresse de l´infertilité; cela peut être une histoire personnelle, l´histoire d´un proche ou de la famille. En pratique on note plusieurs modes de recrutement pour initier une démarche chez les donneurs (ses) de gamètes, qui agissent par altruisme, par solidarité, vis-à-vis des couples infertiles et pour des raisons pécuniaires [23]. Une autre stratégie pour augmenter le nombre de donneur de gamètes est de rémunérer les donneurs. Mais la notion d´indemnisation crée de grandes divergences. Cette pratique soulève une question éthique. Le corps humain ou tout produit du corps humain est hors-commerce, on ne peut le vendre ou l´acheter. La gratuité du don laisse à chaque personne sa pleine liberté de décision, sans pression ni contrainte.

Même si l´AMP avec don de gamètes est connue, elle pourrait ne pas être la seule possibilité devant une infertilité des couples. Ainsi différentes alternatives comme l´adoption pleine, ou l´adoption traditionnelle (vidomégon), s´offrent aux couples infertiles qui ne veulent pas ou qui ne peuvent pas bénéficier de l´AMP par le don de gamètes.

La principale limite de notre étude résulte du choix de la population d´étude. Une étude menée en population générale générale aurait certainement permi de mieux cerner cette problématique dans la population béninoise.

## Conclusion

La prise en charge de l´infertilité grace à l´AMP avec le don de gametes est une problématique peu connue des étudiants en Médecine de Cotonou. Aussi, les dispositions médicale et légale sont peu maitrisées par notre population cible. Au Bénin, la connaissance des étudiants sur l´AMP est correcte, mais le refus de faire le don de gamètes est très important et la principale raison du refus est l´éthique.

### Etat des connaissances sur le sujet

- *Les indications de l´AMP nécessitant le don de gamètes;*
- *Les raisons motivant les individus a donner leur gamète sont bien connues dans les pays développés*.

### Contribution de notre étude à la connaissance

- *Notre étude aborde pour la première fois la problémtique du don de gamètes en milieu africain;*
- *Nous avions donc mis en exergue les différentes motivations de l´acceptation/refus du don de gamètes au sein d´une population cible*.

## Remerciements

Les auteurs remercient tous les étudiants ayant participé à cette enquête.
